# Soft tissue non-Hodgkin lymphoma of shoulder in a HIV patient: a report of a case and review of the literature

**DOI:** 10.1186/1477-7819-6-111

**Published:** 2008-10-21

**Authors:** Domenico Marotta, Alessandro Sgambato, Simone Cerciello, Nicola Magarelli, Maurizio Martini, Luigi Maria Larocca, Giulio Maccauro

**Affiliations:** 1 Department of Orthopedics and Traumatology, Università Cattolica del Sacro Cuore, Rome – Italy; 2 "Giovanni XXIII" Cancer Research Center – Università Cattolica del Sacro Cuore, Rome – Italy; 3 Department of Radiology, Università Cattolica del Sacro Cuore, Rome – Italy; 4 Department of Pathology, Università Cattolica del Sacro Cuore, Rome – Italy

## Abstract

**Background:**

The risk of developing lymphoma is greatly increased in HIV infection. Musculoskeletal manifestations of the human immunodeficiency virus (HIV) are common and are sometimes the initial presentation of the disease. Muscle, bone, and joints are involved by septic arthritis, myopathies and neoplasms. HIV-related neoplastic processes that affect the musculoskeletal system include Kaposi's sarcoma and non-Hodgkin's lymphoma, the latter being mainly localized at lower extremities, spine and skull.

**Case presentation:**

The Authors report a case of a 34 year-old lady. In December 2003 the patient noted a painless mass on her right shoulder whose size increased progressively. In March 2004 she was diagnosed HIV positive and contemporary got pregnant. The patient decided to continue her pregnancy and to not undergo any diagnostic procedure and treatment. At the end of August she underwent a surgical ablation of the lesion that revealed a lesion of 7 cm × 7 cm × 3,3 cm. The histology showed B-cells expressing CD20, PAX-5, CD10, BCL-6 and MUM-1 with 70% Ki67 positive nuclei. The lesion was also negative for EBV infection and showed a monoclonal rearrangement of IgH chain and a polyclonal pattern for TCR gamma and beta. A final diagnosis of diffuse large B-cell lymphoma was made. The patient underwent postoperative chemotherapy. At four-years follow up the patient is symptom free and no local nor systemic recurrence of pathology has been noted on MRI control. HIV infection is still under control.

**Conclusion:**

In this report, we present a case of diffuse large B-cell lymphoma localized in the soft tissue of the shoulder in a HIV infected patient. Authors want to underline this case for the rare position, the big size and the association with HIV infection.

## Background

The risk of developing lymphoma is greatly increased in HIV infection which induces a severe impairment of the immune system due to the progressive reduction of CD4 lymphocytes thus leading to the development of different infections and tumors. The improvement of the therapy with longer life expectancy has led to new associations and, among these, the involvement of other tissues such as the musculoskeletal system. Usually musculoskeletal lesions involve lower limb. Authors report a rare case of soft tissue lymphoma localized at right shoulder in a HIV-infected patient.

## Case presentation

**Figure 1:**
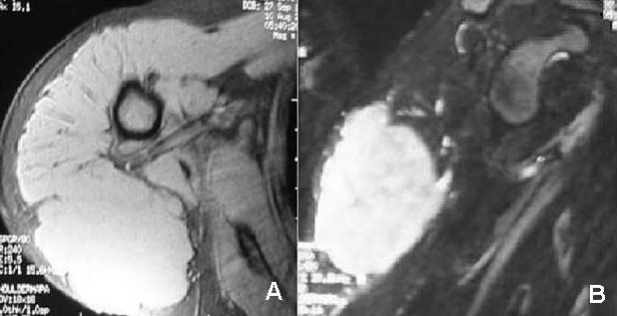
**MRI images of the lesion.** A) Axial MRI performed with T1 gradient-eco sequence. B) TSE sequence with deletion of the T2 signal from adipose tissue.

A 37 years old lady (who was born in 1971) underwent a surgical goldbladder ablation and a excision of appendicitis in 2001. Two years later, in December 2003 the patient noted a painless mass on her right shoulder increasing progressively with time. Her last menstrual cycle was dated in November 2003. In March 2004 she was diagnosed with HIV and at the same time she started her pregnancy. At that time her blood count showed: WBC 6250, lymphocytes 680, CD4 184 (27%) and CD8 354 (52%), and analysis of HIV type 1 RNA detected 975 HIV RNA copies/ml. Even if the lesion kept on growing fast, the patient decided not to undergo any diagnostic procedure nor any possible treatment until the end of pregnancy. In the last period she suffered a mild diabetes, anyway in July gave birth with no further problems. Then she performed a MRI which showed, in T1W sequence, an homogeneous isointense lesion in the posterolateral aspect of the right shoulder below the deltoid muscle (Figure 1A). The TSE sequence with deletion of the T2 signal from adipose tissue showed a marked homogenous hyperintense signal. The margins appeared clean and regular in the absence of any evidence of infiltration, bone lesions and bone marrow involvement (Figure 1B).

At the end of August she underwent a surgical excision of the lesion. The procedure was performed under general anesthesia through a posterior incision. The split of the deltoid fibers revealed a large lesion (7 cm × 7 cm × 3,3 cm) which was excised with free margins.

**Figure 2:**
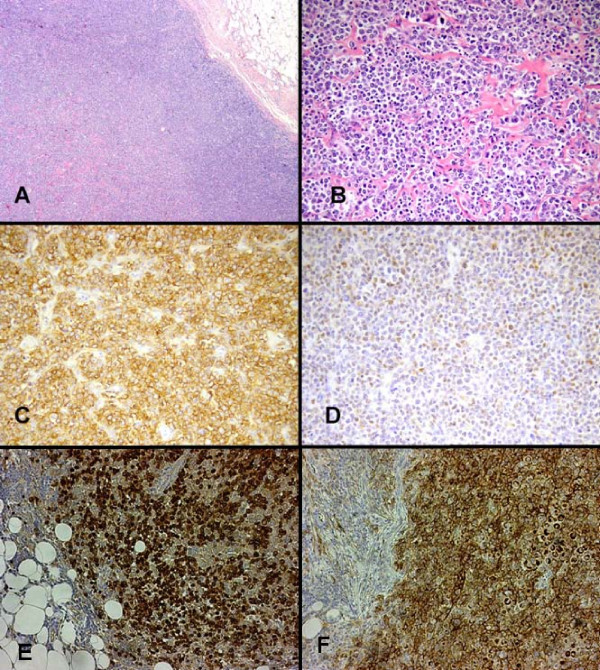
**Hematoxylin and eosin (A, B) staining of the lesion and representative immunostaining images (C-F).** Shown are immunostatining examples of CD20 (C), Bcl-6 (D), PAX-5 (E) and CD10 (F). Original magnification: ×100 (A) and ×250 (B-F).

**Figure 3:**
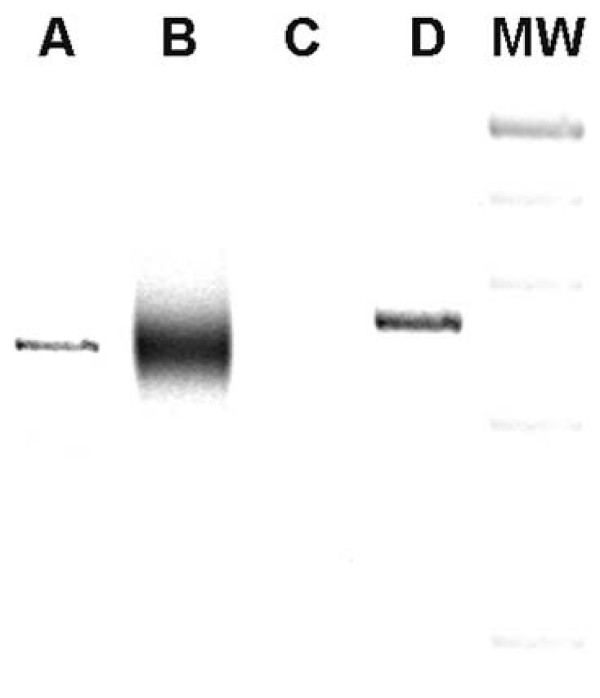
**Molecular analysis for IgH chain rearrangement (FR2 region).** A monoclonal pattern is evident in the analyzed sample (*Lane A*) as well as in the control for a monoclonal pattern (Raji cell line) (*Lane D*). Also shown are the negative control (water) (*Lane C*), a control for a polyclonal pattern (reactive lymphoadenitis)(*Lane B*) and the molecular weight marker (*Lane MW*).

The histology showed a diffuse proliferation of large lymphoid cells with irregular round or oval nuclei. For immunophenotypic studies the avidin-biotin-peroxidase complex (ABC) method was performed on paraffin sections using a commercially available kit (Dako LSAB 2; Dakopatts, Golstrup, Denmark) and the following commercially available monoclonal antibodies: CD3, CD10, CD20, PAX-5, BCL-6, CD138, MUM-1 and Ki67. EBV infection was evaluated by in-situ hybridization of EBV-encoded small RNAs (EBERs) on formalin-fixed, paraffin-embedded tissue sections. In-situ hybridization analysis was performed using a cocktail of fluorescein-isothiocyanate-labeled oligonucleotides complementary to the nuclear EBER RNAs, following the manufacturer's instructions (Dako; Dakopatts, Golstrup, Denmark), as previously described [1]. Neoplastic cells were CD20, PAX-5, CD10, BCL-6, and MUM-1 positive with 70% Ki67 positive nuclei (Figure 2). EBV infection (in-situ hybridization) was negative (data not shown) [1]. Molecular analysis for clonal rearranged immunoglobulin (Ig) and T-cell receptor (TCR) gamma and beta (performed following the multiplex PCR assays and protocols of BIOMED-2 collaborative study) [2] showed a monoclonal rearrangement of IgH chain (Figure 3) and a polyclonal pattern for TCR gamma and beta (data not shown). A final diagnosis of diffuse large cells B-lymphoma was made.

The patient underwent postoperative chemotherapy according with CHOP (cyclophosphamide, doxorubicin, vincristine, and prednisonesix) regimen. The split of the deltoid muscle allowed a fast and complete range of movement regain. After surgery patient also started HIV treatment with a combination of Combivir (Lamivudine plus Zidovudine) and Kaletra (Lopinavir/ritonavir). At four years follow up the patient is symptom free and no local or systemic recurrence has been noted; HIV infection is still under control.

## Discussion

HIV musculoskeletal manifestations are common and are sometimes the initial presentation of the disease. Muscle, bone, and joints are involved and could be affected by infection (such as tuberculosis, pyogenic infection), arthritis, myopathies, neoplasms and miscellaneous conditions such as avascular necrosis and hypertrophic osteoarthropathy [3].

The association between HIV and lymphomas is relatively common. The most frequent localizations are brain, lung, tonsils and stomach followed by oral mucosa, neck, musculoskeletal, subcutaneous and cutaneous tissues. In about 70% of cases lymphomas are non-Hodgkin (NHL) while only 30% are Hodgkin lymphomas [4,5]. The most frequent histological types are diffuse large B-cell lymphoma (30–40%) and Burkitt's lymphoma (40–50%) [4]. Unusual lymphoproliferative disorders associated with HIV infection are: plasmablastic lymphoma, Castleman disease, EBV-associated lymphoproliferative disorders, T-cell lymphoma and primary CNS lymphoma [6].

The incidence of NHL in HIV patients is 3% and lesions are usually high grade and extra nodal [4,5]. Extranodal NHL of soft tissues is a rare disease and is described in only 0,1% of the cases [7]. Primary bone NHL in the absence of extra skeletal disease has also been reported in HIV patients, involves mainly the lower extremities, spine, pelvis and skull and presents with fever, painful unilateral limb swelling, weight loss and pathologic fracture [8,9]. Muscle lesions, generally associated with bone lesions, are mainly described in the psoas muscle and at the lower extremities [10,11]. Cutaneous B-cell lymphomas have been also described in HIV patients as red skin nodules mostly localized at arms, head and neck and trunk. These lesions usually start from the skin and then involves the underneath subcutaneous tissues without cutaneuos ulceration and are usually smaller in size (max 5–7 mm) [12,13] compared with the case reported in this study.

We report a lesion localized in the soft tissues of the shoulder not involving the bone which is not typical in literature for both the position and the large size. To the best of our knowledge, this is the only case in the literature of such a lesion developed in a HIV patient. The mass grew fast during the nine months of pregnancy that is the time spent between the first clinical presentation and the surgical excision, because of the patient decision to not undergo any diagnostic and therapeutic procedure before delivery.

Different MRI appearances of primary muscle lymphomas have been reported in the literature [14,15]. The mass may appear hyper- or iso-intense on T1W images. Hosona et al reported an homogeneous enhancement in two cases of non-primary muscle lymphomas [16] while Beggs reported six patients in which the mass appeared iso/minimally hyperintense on T1W and became hyperintense on T2W with fat suppression sequences. Infiltration of the subcutaneous fat was a typical feature in these cases [17]. The ultrasound and CT appearances of primary muscle lymphomas are also generally non-specific and compatible with neoplastic or inflammatory diseases [18].

HIV positive patients can develop pyomyositis, polimyositis or muscle lymphomas which may be multifocal and produce bright signals on T2-weighted sequences with fat suppression [19,20]. Tehranzadeh et al. suggested that MRI imaging is important for evaluating bone marrow changes and characterizing adjacent soft-tissue involvement [21]. Bone marrow changes are seen as areas of hypointensity on T1-weighted images and as areas of hyperintensity on STIR images or fat-saturated fast spin-echo T2-weighted images. The associated soft-tissue mass appears hyperintense on T2-weighted images. CT and scintigraphy have a complementary role in evaluating affected patients. Imaging findings are similar to those in osteomyelitis, and clinical correlation is often needed. The biopsy is necessary to define the diagnosis and should be performed in HIV patients to exclude pyomyositis.

Surgery in soft tissue lymphoma is still controversial. Damron et al. are convinced that lymphoma is a non surgical disease in which chemotherapy and/or radiotherapy are adequate therapeutic strategies and they do not recommend the surgical excision since it would remove a clinical barometer of responsiveness to medical treatment. Biopsy should be only performed to confirm the nature of the lesion, especially in differential diagnosis with soft tissue sarcoma [22].

On the contrary Bozas et al. described a case of abdominal wall mass (10 × 18 cm) situated between the abdominal muscles and in which a wide excision was performed followed by immuno-chemotherapy [23]. Belaabidia et al. also described a case of muscle lymphoma of biceps femoris (17 × 14 × 7 cm) in which treatment was wide surgery followed by chemotherapy [24].

No other cases of soft tissue lymphomas of the shoulder in HIV patients with dimensions compared to the one reported in this study have ever been described in the literature. Because of the large dimension, Authors performed an excision biopsy with tumor-free margins followed by chemotherapy. This combined treatment was expected to reduce the risk of local recurrences allowing good functional recovery of the shoulder. Radiotherapy alone without removing the mass was excluded for the high risk of infection. Follow up have confirmed the appropriateness of the treatment since the patient is still disease-free after four years with good range of motion.

## Conclusion

This study reports a very rare localization of a case of diffuse large B-cell lymphoma in the soft tissue of the shoulder in a HIV infected patient. Excision biopsy followed by chemotherapy allowed a good local and systemic control of the disease with a good functional recover after four years.

## Consent

Written consent was obtained from the patient for publication of this case report.

## Competing interests

The authors declare that they have no competing interests.

## Authors' contributions

DM prepared the draft of case report. GM conceived the idea of the case report and helped with the draft of it. AS, SC and AD helped the draft of the case report. MM and LMR performed the molecular analyses. NM performed the radiological studies and helped with draft of case report. All authors read and approved the final manuscript.
